# Giant elastic tunability in strained BiFeO_3_ near an electrically induced phase transition

**DOI:** 10.1038/ncomms9985

**Published:** 2015-11-24

**Authors:** Q Li, Y. Cao, P. Yu, R. K. Vasudevan, N. Laanait, A. Tselev, F. Xue, L. Q. Chen, P. Maksymovych, S. V. Kalinin, N. Balke

**Affiliations:** 1 Center for Nanophase Materials Sciences and Institute for Functional Imaging of Materials, Oak Ridge National Laboratory, Oak Ridge, Tennessee 37831, USA; 2 State Key Laboratory for Low-Dimensional Quantum Physics, Department of Physics and Collaborative Innovation Center for Quantum Matter, Tsinghua University, Beijing 100084, China; 3 RIKEN Center for Emergent Matter Science (CEMS), Wako, Saitama 351-0198, Japan; 4 Department of Materials Science and Engineering, Pennsylvania State University, University Park, Pennsylvania 16802, USA

## Abstract

Elastic anomalies are signatures of phase transitions in condensed matters and have traditionally been studied using various techniques spanning from neutron scattering to static mechanical testing. Here, using band-excitation elastic/piezoresponse spectroscopy, we probed sub-MHz elastic dynamics of a tip bias-induced rhombohedral−tetragonal phase transition of strained (001)-BiFeO_3_ (rhombohedral) ferroelectric thin films from ∼10^3^ nm^3^ sample volumes. Near this transition, we observed that the Young's modulus intrinsically softens by over 30% coinciding with two- to three-fold enhancement of local piezoresponse. Coupled with phase-field modelling, we also addressed the influence of polarization switching and mesoscopic structural heterogeneities (for example, domain walls) on the kinetics of this phase transition, thereby providing fresh insights into the morphotropic phase boundary in ferroelectrics. Furthermore, the giant electrically tunable elastic stiffness and corresponding electromechanical properties observed here suggest potential applications of BiFeO_3_ in next-generation frequency-agile electroacoustic devices, based on the utilization of the soft modes underlying successive ferroelectric phase transitions.

## Abstract (web summary)

Ferroelectric materials possess spontaneous electrical polarization coupled to their underlying lattice structure, which may be utilized technologically. Here, the authors use band-excitation piezoresponse/elastic spectroscopy to study the sub-megahertz dynamics of a structural phase transition in BiFeO_3_.

## Graphical abstract

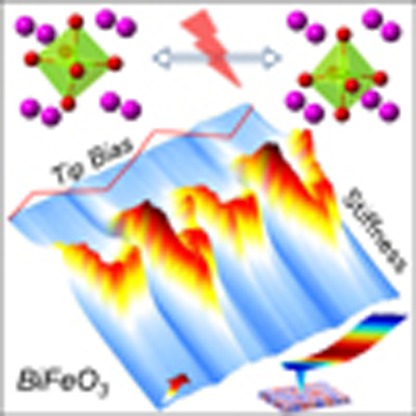

Ferroelectrics underpin a vast spectrum of modern technologies, including ultrasonic transducers, precision actuators, nonvolatile memories, to name a few^1^,^2^,^3^. Pivotal to the continued development of ferroelectrics is a deep understanding of their structural−dynamics relationship, which is conventionally separated into lattice and domain structure levels^4^. The intrinsic lattice contribution has long been centred on the concept of morphotropic phase boundary (MPB), which describes the degeneracy/coexistence of, classically, rhombohedral (**R**) and tetragonal (**T**) phases across structural transitions driven by composition^5^, temperature^6^, applied electric (E-) field^7^, epitaxial strain^8^,^9^ and pressure^10^,^11^. This MPB picture has been substantially refined over last two decades through the discoveries of monoclinic bridge phases^12^,^13^ and associated polarization (P) rotation mechanism^14^. At the domain level, extrinsic structural dynamics takes place via domain wall motions and contribute to many of the ferroelectric properties^15^,^16^. Recent studies have also revealed that monoclinic phases can be stabilized under local stress/electric fields generated by complex domain structures in BaTiO_3_ and KNbO_3_ (refs 6, 17), thus blurring the boundary of such structural hierarchy.

Scattering techniques based on X-ray/neutron and electron probes are capable of resolving subtleties in crystal structure and lattice dynamics near the MPBs and allow *in situ* tracking of their response to external stimuli; however, these techniques generally fall short of capabilities to correlate structural dynamics with real-space microstructures, especially regarding domain structures. To this end, piezoresponse force microscopy (PFM) has emerged as a primary tool for imaging electromechanical properties of materials with nanoscale resolution^18^, and PFM spectroscopy has also been developed in some forms enabling local probing of polarization switching dynamics^19^,^20^,^21^. This latter issue has been the subject of extensive PFM studies, yielding significant observations of, for example, low-energy nucleation centres at ferroelastic domain walls^20^,^22^. In a limited number of cases, hints of phase transition phenomena have also been reported^9^,^23^.

As the most studied multiferroic compound, BiFeO_3_ (BFO) shows many promising functionalities emerging from its intercoupled polarization, transport, mechanical and magnetic properties^8^,^9^,^22^,^24^,^25^,^26^,^27^,^28^. Mixed-**R**/**T** phase BFO thin films near a strain-induced MPB (on LaAlO_3_ substrates) have attracted particular interests for piezoelectric applications due to their large field-induced strains and shape-memory effects^8^,^9^,^29^,^30^,^31^,^32^,^33^. These effects, though being reversible, are hysteretic (that is, the field-induced state has to be annihilated by applying an opposite field) as a consequence of the intricate mixed-phase microstructure and thus are undesirable in applications of, for example, tunable devices^3^. For such purpose, BFO in the stability field of **R**-phase is to be considered instead.

Here, we employed band-excitation^34^ piezoresponse spectroscopy (BEPS) to probe local bias-induced **R**−**T** phase transition as well as polarization switching behaviour of strained **R**-BFO thin films. By deciphering the elastic spectroscopy inherent in BEPS that acts as a nanoscale analogy to macroscopic resonant ultrasound techniques^35^, we examined the sub-MHz elastic dynamics associated with both intrinsic and extrinsic structural activities of the system. This added information channel supported by phase-field modelling allowed us to further explore the kinetic interaction between the polarization switching and heterogeneous domain structures with the **R**−**T** transition. Our results highlight giant electrically tunable elastic properties of BFO heretofore largely unappreciated.

## Results

### Bipolar BEPS loops

**Figure 1:**
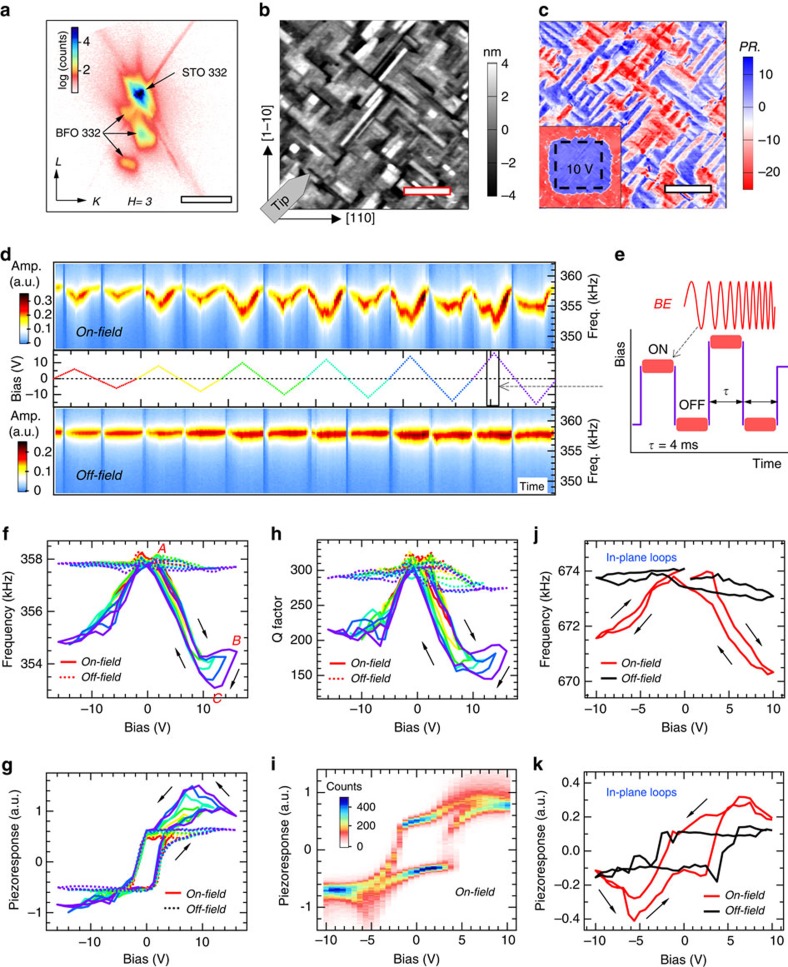
Bipolar BEPS switching loops of 50 nm BFO/STO. (**a**) Reciprocal space map near the STO 332 reflection showing three-fold splitting indicative of a **M**_**A**_ structure of BFO. Scale bar, 1/3.905 Å^−1^. (**b**,**c**) Morphology (**b**) and corresponding lateral PFM (**c**) images; Inset in **c** shows the corresponding vertical PFM image after the center square area was poled with 10 V tip bias. The cantilever alignment adopted in all BEPS measurements is shown in **b**. Scale bar, 1 μm. (**d**–**h**) Single-point BEPS performed under ascending bias windows as shown in **d**, which also illustrates the measured piezoresponse amplitude spectrographs; total time length=2.3 s. **e** is a schematic representation for the chirp-type BE waveform and on/off-field d.c. switching sequences. **f**–**h** are the fitted frequency (**f**), piezoresponse (**g**) and Q factor (**h**) loops with their sense marked by arrows and colour coded by the bias windows shown in **d**. (**i**) Histogram of the on-field piezoresponse loops acquired from 50 × 50 points over a 1 × 1 μm^2^ region. (**j**,**k**) Single-point in-plane BEPS frequency (**j**) and piezoresponse (**k**) loops acquired in the lateral mode with the sense marked by arrows. a.u., arbitrary unit.

In this study, we focus on a 50 nm BiFeO_3_ thin film with a bottom layer of La_0.7_Sr_0.3_MnO_3_ grown on (001)-SrTiO_3_ (STO) substrates (cubic indices are used in this article). The as-grown BFO film comprises four essentially equally populated, upwards-polarized twin variants due to −1.5% epitaxial strains imposed by STO and certain electrical boundary conditions^36^,^37^. X-ray reciprocal space mapping confirms a distorted **R** (or monoclinic **M**_**A**_ (ref. 13); Fig. 1a) average symmetry of the film with *c*/*a* axis ratio=1.043. Figure 1b,c illustrates the surface morphology and corresponding vertical/lateral PFM images showing mutually orthogonal surface domain patterns in widths of ∼100 nm and separated by largely straight boundaries. Figure 1d–h presents a typical set of single-point (out-of-plane) BEPS results acquired at the domain interior. As is readily seen from the amplitude spectrographs (Fig. 1d), the on-field response shows strong shifts in contact resonance frequency (*f*_c.r._) during all the switching cycles, in striking contrast with the off-field response. Further details can be extracted from the derived loops: the on-field *f*_c.r_ shifts downwards with bias in both polarities and for large bias windows, it saturates (and slightly recovers) before the maximum bias followed by a second drop when decreasing the bias thus forming characteristic hysteresis (see the branch *A*–*C* in Fig. 1f); for the off-field *f*_c.r_ loops, only weak anomalies can be found near ∼±3 V where the domain switching events occur^38^. Similar patterns are also followed by the quality (*Q*)-factor loops with relatively larger variation in the off-field state (Fig. 1h).

The off-field piezoresponse loops have the canonical square shape gradually saturating under positive tip biases^19^ (Fig. 1g). By comparison, the on-field loops show bias-dependent, enhanced piezoresponse in accordance with the shift patterns of *f*_c.r_; in particular, a ‘head'-like hysteretic feature forms in the positive half-cycle. Along with the *f*_c.r_ anomalies, this feature is well reproducible in BEPS mapping across the film on average leading to two- to threefold enhancement (versus zero-bias) of piezoresponse, and its propensity appears to be weaker for the negative bias side where the applied E-field is parallel to the pristine *P* direction (Fig. 1i). Note that the measured on-field piezoresponse in these cases is truly dominated by electromechanical response over weak or no contributions from the parasite electrostatic and conduction current effects (Supplementary Note 1 and Supplementary Figs 1,2). This loop shape is also distinct from those peculiar backswitching loops mediated by charge injection, since it occurs only on-field and without any self-crossings^39^.

In BEPS, systematic and reproducible changes in *f*_c.r_ are due to alteration of the elastic properties of samples^38^, as is indeed the case here. Through cantilever dynamics and contact mechanics calculations (see details in Supplementary Note 2 and Supplementary Table 1), we find that the Young's modulus (*Y*) of the BFO film changes from ∼204 GPa in the pristine state to, for example ∼141 GPa at 12 V shown in Fig. 1f, where the Δ*f*_c.r_ peaks at ∼4.7 kHz, amounting to a remarkable ∼30% elastic softening. This softening behaviour can be instantaneously switched on/off by bias within the probed millisecond timescale, and remains basically unchanged when the contact resonance is driven by external mechanical excitation^38^. Similar softening effects are also observed in the lateral/torsional BEPS mode, where in-plane polarization is detected and torsional *f*_c.r_ reflects the shear modulus (*G*) of samples^40^. Overall, the lateral piezoresponse loops of this BFO film are not well-defined in the low-bias region compared with their simultaneously acquired out-of-plane counterparts, presumably due to its complex in-plane switching behaviour; however, there is a recurring feature manifest in all the in-plane loops: as illustrated in Fig. 1j,k, the on-field piezoresponse is enhanced along with the elastic softening up to a threshold bias and then drops quickly. All these observed BEPS characteristics imply an intrinsic dynamic process taking place, reminiscent of the **R**−**T** phase transition of the system. The measured *Y* variations of the BFO/STO film are also found to be bounded by the elastic moduli of different **R-** or **T**-phase BFO samples probed at GHz frequencies by Doig *et al*^41^. Note that similar frequency response and piezoresponse enhancement were also observed in (001)-BFO thin films under different epitaxial strains (for example, BFO/DyScO_3_, strain approximately −0.5%; Supplementary Note 3 and Supplementary Fig. 3).

### Phase-field modelling of a single domain

**Figure 2:**
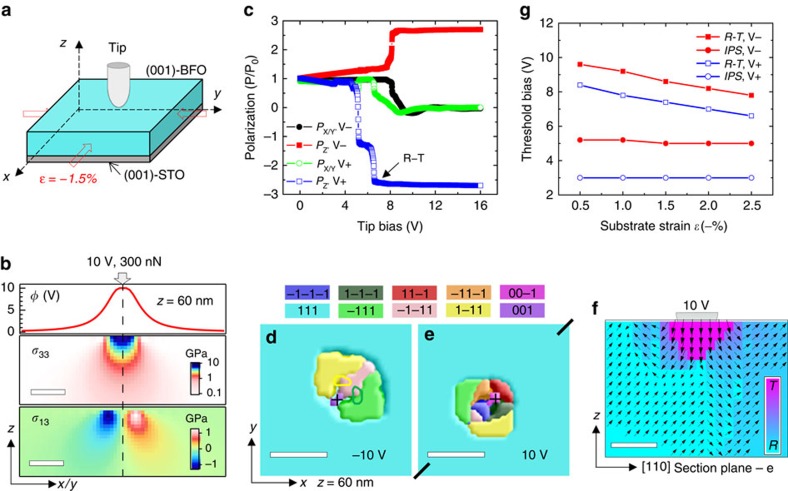
Phase-field modelling of a single-domain BFO. (**a**) Schematic for the model geometry (not to scale): *x*=*y*=128 nm, *z*=50/10 nm, tip coordinate=(64, 64, 60) nm. Thick arrows represent the biaxial substrate strain *ɛ*=*–*1.5%. (**b**) Line/plane distribution profiles of the tip potential *φ* and normal/shear stress components *σ*_33_/*σ*_13_ under 10 V tip bias and 300 nN loading force. Scale bar, 10 nm. (**c**) Evolution of local polarization *P*_(*X*, *Y*, *Z*)_ components underneath the tip during incremental application of positive (V+) and negative (V−) biases. *P*_0_=0.58 C m^−2^. (**d***–***f**) Stabilized domain/phase configurations at selected biases. In **d**,**e**, the *P* direction is color-coded and the tip center position marked by a cross. The outlines of the nucleated [1–11]_***R***_ and [−111]_***R***_ domains at *–*5 V are overlaid on **d**. Arrows in **f** show the direction of *P* and scale with its magnitude in size. Scale bar, 20 nm in **d**,**e** and 5 nm in **f**. (**g**) Calculated in-plane switching (IPS) and **R**−**T** phase transition threshold biases as a function of substrate strain.

Phase-field modelling offers direct visualization of the evolution of ferroelectric polarization during BEPS measurements. We start from a single [111]_***R***_-domain BFO/STO model as illustrated in Fig. 2a. Here the lateral tip potential spreading is modelled as a flat-top Lorentz function, *φ*(*x*,*y*)=*φ*_0_*γ*^2^/((*r*−*a*)^2^+*γ*^2^),*r*>*a*;=*φ*_0_,*r*≤*a*, where *r* is the distance from the tip and *γ* is the half width at half maximum of applied bias, *φ*_0_; given the presence of strong tip indentation^42^, the center area with radius *a*, obtained self-consistently from the contact mechanics calculations, is set equipotential. Figure 2b shows the calculated depth profiles of normal and shear stresses under the tip loading force. Both types of stresses are concentrated within ∼10 nm length scale, indicating the size of sample volume where local elasticity is effectively probed^40^, approximately commensurate with that for piezoresponse. Figure 2c shows the calculated local *P* values underneath the tip following increasing sequences of tip bias. For both bias polarities, there are sudden jumps in *P* occurring within 6–10 V, after which its *Z* component, *P*_*Z*_ increases to ∼2.7*P*_0_ and the *P*_*X*_/*P*_*Y*_ components reduce to zero. Such jumps signify the **R**−**T** phase transition and the calculated *P*_*Z*_ faithfully reproduces the experimental *P* value (∼1.5 C m^−2^) of **T-**BFO^43^. The fact that this transition occurs earlier by ∼2 V along the antiparallel E-field direction (positive bias) is somewhat counterintuitive, as one may assume that the **M**_**A**_−**T** polarization rotation path would be ‘shorter' in the parallel E-field condition^14^. This points to a competing interaction between the polarization switching and rotation processes, as the domain/phase configuration results from minimization of the system free energy with respect to the driving force, electrostatic energy −EP. The stabilized domain configurations under ±10 V are presented, for example, in Fig. 2d–f. Noticeable in-plane switching (IPS) activities can be seen for both polarities (for positive tip biases, the IPS also involves out-of-plane switching) due to the lateral components of tip E-fields^22^ (Supplementary Fig. 4 and Supplementary Note 4), leading to ∼20 nm domain clusters with four **R-**phase twins enclosing a **T-**phase domain underneath the tip (Fig. 2d,e). In the case of 10 V, the **T-**phase extends ∼4 nm into the film assuming a wedge-shape depth profile and around it, a thin (1 or 2 nm) transition layer with higher *P* than that of **R-**BFO is also visible (Fig. 2f), which could be an indicator of the **R**/**T** phase boundary thickness^8^. Furthermore, we considered the influence of substrate strain on the **R**−**T** transition as well as IPS of the **R-**phase. Here the IPS threshold bias is defined as the voltage at which nascent domains are visually detected on the surface, since they do not nucleate right below the tip (see an illustration on Fig. 2d). Within the strain range from −0.5% to −2.5%, the **R**−**T** threshold bias decreases monotonically as expected, while that of IPS appears almost independent of substrate strain (Fig. 2g).

In light of these modelling results, we attribute the observed BEPS features to the occurrence of the **T**-phase. Note that **T**-BFO is not soft *per se*, neither in terms of elasticity nor piezoelectricity^44^. Therefore, the softening behaviour has to be explained by the **R**/**T** phase coexistence (that is, MPB) or more naturally, the **R**−**T** phase instability^4^,^45^. Elastic softening occurs near phase transitions following specific patterns as determined by their transition characters, for example, order parameter type^46^. In our case, it is more complicated because the transition is locally and non-uniformly driven; therefore, the measured dynamics is a convolution of the lattice dynamics and the growth kinetics of the emergent phase. For instance, when a **T**-phase nucleus grows outwards under higher biases, the inner region recovers as it passes through the instability field but the surrounding lattice enters that and softens overall, probably maintaining the softening trend for the probe volume. This renders it not straightforward to assign an exact **R**−**T** transition point to the measured elastic or piezoresponse spectra, albeit their peak softening biases compare favourably with the calculated threshold biases. In this regard, the lateral piezoresponse may be more informative, since in principle it changes most drastically during the **R**−**T** transition.

### Unipolar BEPS and strain loops

**Figure 3:**
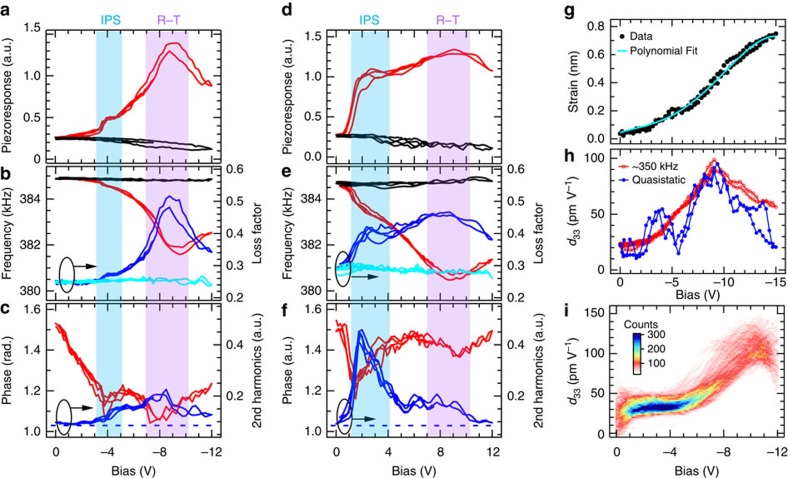
Unipolar BEPS switching loops of 50 nm BFO/STO. (**a**–**f**) Unipolar BEPS loops acquired under negative (**a**–**c**) and positive (**d**–**f**) tip biases at the same location. The on-field loops are in red/blue and the off-field loops in black/cyan. Light blue and purple shadowed areas are guides to eyes indicating the in-plane switching (IPS) and **R**−**T** transition regions, respectively. Dashed lines in **c**,**f** denote the second harmonics response signal noise floor. (**g**,**h**) Single-point strain curves (**g**) and its derivative (that is, static *d*_33_) loops versus corresponding on-field piezoresponse loops (**h**). (**i**) Histogram of the static *d*_33_ loops calculated from 40 × 40 strain curves acquired over a 1 × 1 μm^2^ region, via a 6th-order polynomial fitting to the measured data as illustrated in **g**. a.u., arbitrary unit.

To further find links between the softening behaviour and the IPS and **R**−**T** transition of the BFO film, we performed unipolar BEPS in the parallel E-field condition; to fulfil this, a pre-poling cycle was adopted in the positive bias case so as to create a single-*P*_Z_ domain state. In contrast to the bipolar loops, most unipolar loops measured off the pristine domain boundaries are essentially non-hysteretic and undergo a continuous softening to the deepest point (as would be the point-*C* in Fig. 1f), followed by a recovery or hardening when the **T**-phase continues to grow with bias. A typical set of single-point unipolar loops are presented in Fig. 3a–f. These loops clearly illustrate two anomalies associated with both types of structural activities. The IPS occurs in the low-bias regions close to the modelling results, and in this case (and many other measured points), it is much stronger for positive tip biases as is reflected by a marked enhancement of piezoresponse (*cf.* Fig. 3a,d), which is also accompanied with large increases in *Q*^−1^ (Fig. 3b,e), the loss factor signifying internal energy dissipation, as well as in second harmonic piezoresponse (Fig. 3c,f). Previous studies have established that the second harmonic piezoresponse of ferroelectric materials mainly originates from reversible interface (domain walls and/or phase boundaries) motions under a.c. excitations^47^,^48^. Therefore, all these observations lead to a coherent picture of enhanced domain wall motions contributing extrinsically to the piezoresponse near the IPS coercive E-fields. On the other hand, the IPS seems only to cause moderate perturbation to the smooth, wide-range operating elastic softening driven by the **R**−**T** phase instability, although it can increase the piezoresponse and energy loss to a much higher degree. This agrees with macroscopic elastic studies in which the influence of mobile twin walls appears more noticeably as damping than reduction in the apparent elastic moduli of samples in the same sub-MHz frequency range^35^. Such difference can be greater in BEPS presumably due to its highly localized, weak excitation nature and thus the probed real part elasticity is essentially intrinsic. At the **R**−**T** transition regions, the piezoresponse, Δ*f*_c.r_ and *Q*^−1^ all reach their maxima; however, these anomalies along with those in the phase signals are not exactly synchronized in bias, implying subtle difference in their sensitivities to structural changes. The dominant piezoresponse enhancement and dissipation mechanisms at this stage should be attributed to the intrinsic lattice softening of BFO, as is also corroborated by the weak second harmonic piezoresponse measured here that evidences insignificant interphase boundary mobility.

We also acquired static on-field strain curves simultaneously with unipolar BEPS, as illustrated by typical single-point results in Fig. 3g,h. Near the **R**−**T** transition, the piezoelectric coefficient (*d*_33_) derived from the strain curves appears basically the same as the dynamic piezoresponse probed at ∼350 kHz within the calibration accuracy, again implying that the piezoresponse is intrinsic here. Besides that, the strains curves exhibit stronger fluctuations around the IPS region, which can be expected as a result of higher twin wall mobility under slowly varying E-fields. Figure 3i shows the histogram of *d*_33_ loops derived from a mapping data set of strain curves based on their polynomial fits (for the noise reduction purpose; Fig. 3g). The peak *d*_33_ of these loops distributes over 100−130 pC/N, apparently comparable to that attained in mixed-phase BFO films^9^.

### Effects of the complex domain structure

**Figure 4:**
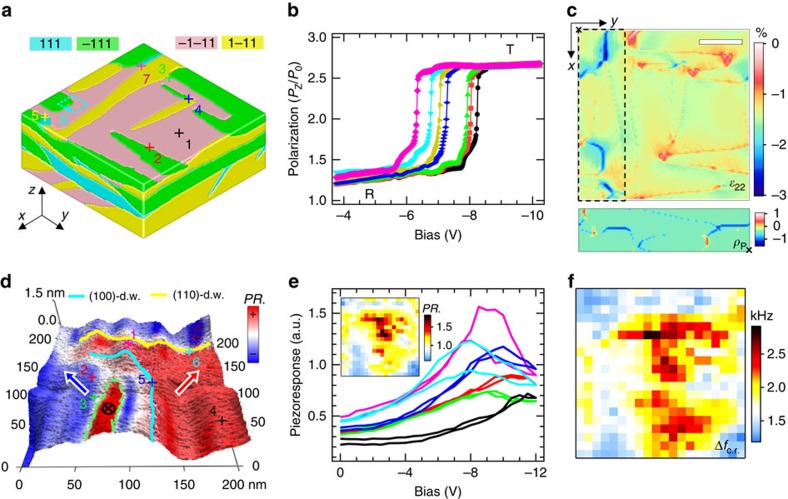
Effects of the complex domain structure of BFO. (**a**) A phase-field simulated four-variant domain structure model (size: 128 × 128 × 50 nm^3^) for **R**-phase BFO/STO thin films. (**b**) Calculated bias evolution of local polarization component *P*_*Z*_ underneath the tip, at various surface positions marked by the crosses in **a** and color-coded accordingly. (**c**) Distribution profiles of the in-plane strain component *ɛ*_22_ and polarization charge ρ_P_ (only for the dashed-line enclosed region; unit: 10^9^ C m^−3^) for the pristine domain structure. Scale bar, 32 nm. (**d**) Lateral PFM domain image (200 × 200 nm^2^) of a domain junction region, on which a 20 × 20 BEPS mapping data set was acquired. Thick arrows denote the in-plane *P* vectors; the whole region has an upwards out-of-plane *P* direction except for a stripe region enclosed by green dashed lines. The cyan and yellow lines denote the surface traces of (100)- and (101)-domain walls, respectively. (**e**) Selected on-field piezoresponse loops acquired at the positions marked in **d** and color-coded accordingly; a map of the maximum piezoresponse is shown in the inset. (**f**) Map of the derived maximum resonance frequency shift, Δ*f*_c.r_. a.u., arbitrary unit.

We have shown that the kinetic interaction between polarization switching and the **R**−**T** transition, well within the domain interior, causes characteristic asymmetry and hysteresis in the elastic softening patterns of the BFO. Next, we examine the effects of the heterogeneous domain structure. Figure 4a is a simulated multi-domain model evolving from random *P* values (only with the *P*_*Z*_ component set positive) under the constraint of −1.5% substrate strains, closely resembling those observed PFM domain patterns. Negative tip biases were applied on a number of surface positions, and the resultant P–V curves for selected points are shown in Fig. 4b. As can be seen, the **R−T** transition starts earlier on domain walls or junctions than in the domain interior with up to ∼2 V difference, and consequently, at the same bias the nascent **T**-phase is more visible on the resultant domain configuration for those ‘easier' regions (not shown). Examination of the pristine strain state (Fig. 4c) reveals that those ‘easiest' regions (for example, p6 and p7) are associated with larger local in-plane compressive strains (than –1.5%, the homogeneous value) as well as larger out-of-plane tensile strains; that is, they are closer to the **T**-phase originally. Across these walls, the polarization vector aligns tail-to-tail leading to an accumulation of bound charge, in agreement with an earlier microscopic observation of **T**-like structure near the charged domain walls in **R**-BFO^49^. Note also that the **R**−**T** transition is slightly easier even on the domain walls with reduced in-plane strains (*c.f.* p3 and p1) suggesting additional roles of domain walls in promoting the **T**-phase growth kinetics.

To confirm these modelling results experimentally, high-resolution BEPS mapping was performed over a typical domain junction, as shown in Fig. 4d, where the domain wall orientations are inferred from the measured PFM images based on the established domain geometry rules^37^. This junction may well correspond to the p6 region in Fig. 4a, and here a narrow stripe of downwards polarized domain running parallel to the (100)-domain wall is observed to have formed to partially compensate the high-density negative polarization charge nearby. Around such locally stress/charge-concentrated domain walls, the higher measured peak softening Δ*f*_c.r_ (Fig. 4f) implies faster **T**-phase growth kinetics than that of the remaining region, consistent with the modelling results. The peak on-field piezoresponse also shows similar contrast in correlation with the elastic softening effect (Fig. 4e); however, this contrast is not fully disclosed in the zero-bias piezoresponse (not shown) as equivalently obtained in static PFM imaging.

## Discussion

For our BEPS measurement geometry, the vertical and torsional contact mode involves the *Y* and *G* moduli, respectively, of the (001)-BFO thin film, corresponding to its elastic compliance or stiffness tensors^50^,^51^: *Y*_[001]_=*s*_11_^−1^=[(*c*_11_−*c*_12_)(*c*_11_+2*c*_12_)]/(*c*_11_+*c*_12_) and *G*=[2(*s*_11_−*s*_12_)]^−1^=(*c*_11_−*c*_12_)/2. Thus, their concomitant anomalies under E-fields appear most likely to be driven by (*c*_11_−*c*_12_). Softening of this elastic constant has been known to signify lattice instabilities against tetragonal shear strains (that is, pointing to the **R**−**T** transition here), common for many displacive ferroic systems as exemplified by Pb-based perovskite relaxors^11^,^46^. For instance, an entire softened acoustic phonon branch propagating along [110] (with [1–10] transverse polarization), controlled by the (*c*_11_−*c*_12_) mode, was observed in near-MPB **R**-phase Pb(Zn_1/3_Nb_2/3_)O_3_−4.5%PbTiO_3_ coinciding with its colossal piezoelectricity^52^. Enhanced thermal diffuse scattering as a clue for *Γ*-point phonon softening was also observed in BFO approaching to an E-field-driven **R**−**T** transition^53^. It is noteworthy that structurally the **T**-BFO stabilized on LaAlO_3_ exhibits a large distortion of the oxygen octahedron and loss of its rotation pattern in **R**-BFO^8^,^43^,^54^, implying that this transition involves multiple lattice instabilities at the *R*/*M*-points. These zone-boundary antiferrodistortive modes, in fact, have been argued based on first-principle calculations to be the primary order parameter, as opposed to the *Γ*-point polar mode, for the **R**−**T** transition of strained BFO^55^, posing it as a (pseudo-)proper ferroelastic transition. All these complex transition characters clearly have effects on the strain-coupling mechanism and elastic softening dynamics of BFO. Given a dearth of experimental lattice dynamics studies of the system thus far, we leave this as an inconclusive account of the detailed microscopic picture behind our observations; nevertheless, these results *per se* are clearly a direct measure of soft acoustic modes of BFO in the long wavelength limit.

This giant elastic softening effect of BFO coupled with non-hysteretic and reasonably low-loss characteristics suggests its usefulness in tunable microwave electroacoustic devices^3^,^56^,^57^,^58^,^59^. Currently, the prevailing materials for these applications are based on paraelectric-phase (Ba,Sr)TiO_3_ with incipient polar instability (that is, soft optic modes), while materials set in polar phases are less often considered due to unfavourable domain switching related hysteresis and loss. In both cases, frequency tunability is underlain by the soft optic phonons (and/or relaxational modes), which harden under d.c. E-fields^56^,^59^, primarily leading to changes in polarization Δ*P*; via an electrostrictive coupling, this Δ*P* shifts the elastic stiffness downwards and upwards for paraelectric and polar phases, respectively, and does so in opposite ways to their electromechanical properties^3^. By contrast, the utilization of BFO here is conceptually different in that the acoustic modes of BFO soften near a successive ferroelectric/-elastic phase transition that is likely triggered by optic soft modes coupled to applied E-fields. Simple estimation suggests that 20−30% Δ*Y*, as we typically observed, can lead to over 10% frequency tunability in a thickness extension-mode resonator. This factor, compared with the state-of-art ∼1−4% values attained in (Ba,Sr)TiO_3_ (refs 3, 58), would make it highly rewarding to explore the demonstration in prototype devices. We further note that the **R**−**T** phase instabilities of BFO can be tailored via epitaxial strain^60^, chemical doping^61^ and possibly a more radical route of superlattice design^59^, and combinations thereof, offering practical ways to circumvent low dielectric strength issues known for BFO^53^.

To realize such functionalities of BFO and materials alike, in turn, requires a deep survey of the structure−dynamics correlation at pertinent length levels thus providing feedback for materials design. The approach here, we demonstrated, effectively bridges the structural gap between the atomic lattice and mesoscopic domains; the complex dynamic interplay between intrinsic order parameters and structural heterogeneity within these length scales necessitates probing their elasticity/polarization dynamics as an integral part. This approach can be immediately extended to other MPB ferroelectric systems. Beyond this, given the ubiquitous elastic anomalies near phase transitions of diverse nature in solid-state materials, our study may also furnish far-reaching clues as to examining their dynamics from the nanoscale under locally or globally applied driving forces (for example, temperature and magnetic fields).

## Methods

### Sample fabrication

50 nm BFO thin films were grown using a reflection high-energy electron diffraction–assisted pulsed laser deposition system on pretreated TiO_2_-terminated STO(001) substrates, with 5 nm per 13 unit-cell La_0.7_Sr_0.3_MnO_3_ as bottom electrodes. The BFO and La_0.7_Sr_0.3_MnO_3_ layers were grown at 690 °C at an oxygen partial pressure of 100 and 300 mTorr, respectively. The laser repetition frequency and energy were set to 1 Hz per 1.5 J cm^−2^ for the growth of La_0.7_Sr_0.3_MnO_3_ and 3 Hz per 2.5 J cm^−2^ for BFO. The crystal structure of the films was examined using X-ray (15.499 keV) diffraction at Beamline 33-BM-C, Advanced Photon Source, Argonne National Laboratory.

### PFM Experiments

PFM was carried out in ambient environment on a commercial atomic force microscope (Cypher AFM, Asylum Research), equipped with a home-developed band-excitation controller based on a National Instruments PXIe-6124 data acquisition card operating in Labview/Matlab software. Pt/Ir-coated conductive Si probes with medium stiffness *k*=2–5 N m^−1^ were used in contact mode with loading forces set at ∼300 nN. The vertical sensitivities of cantilevers were determined from acquired force–distance curves. In BEPS, contact resonance spectra of the tip–sample junction were electrically excited using chirp-type band-excitation signals, typically with 1 V a.c. amplitude, 4–8 ms time length and 40 kHz bandwidth bracketing the resonance peaks, and were captured as a function of d.c. switching waveforms within up to ±16 V bias windows. Measured spectra were fitted to a simple harmonic oscillator model^34^ to decouple intrinsic piezoresponse, resonance frequency (*ω*_*C*_) and *Q* factor with great veracity. The latter two quantities encode near-surface elastic information of the sample; thereby contact resonance plays another important role in BEPS besides signal enhancement. BEPS mapping was performed on grids of points evenly spaced on predefined scan regions. Conduction current was monitored with a current amplifier (DLPCA-200, Femto).

### Phase-field modelling

Temporal, three-dimensional spatial evolution of the ferroelectric polarization of the BFO films is modelled by numerically solving the time-dependent Landau–Ginzburg–Devonshire (LGD) equations^62^,


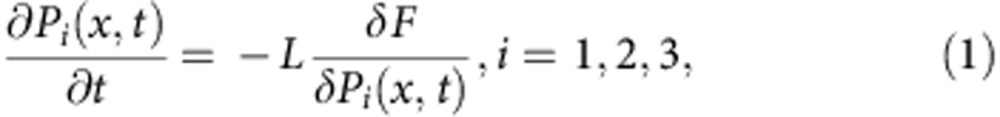


in which *P*_*i*_ is the polarization vector, *x* is the spatial position, *t* is the time, *L* is the kinetic coefficient related to the domain wall mobility, *F* is the total free energy,


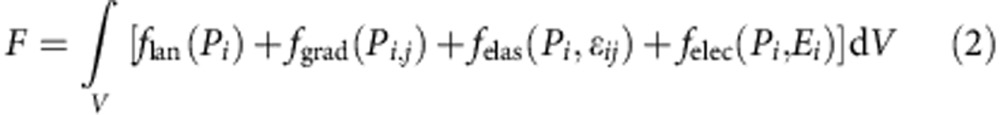


in which *f*_lan_(*P*_*i*_), *f*_grad_(*P*_*i*,*j*_), *f*_elas_(*P*_*i*_, *ɛ*_*ij*_) and *f*_elec_(*P*_*i*_, *E*_*i*_) represent the LGD free-energy density, gradient-energy density, elastic-energy density and electrostatic-energy density, respectively. Detailed description and analytic expressions for these energy terms can be found in literature^63^; in particular, here we adopted an eighth-order LGD free energy expansion for BFO. Isotropic gradient energy coefficients *G*_ii_=0.3 and relative dielectric constant *κ*_ii_=50 were assumed. All the energy coefficients of BFO and detailed model parameters are provided in Supplementary Table 2. Equation (1) was solved by a semi-implicit spectral method^64^ using a realistic three-dimensional geometry sampled on a fine grid mesh of 128Δ*x*_1_ × 128Δ*x*_2_ × 64Δ*x*_3_, where the grid size Δ*x*=1.0 nm. The film and substrate thickness are 50Δ*x* and 10Δ*x*, respectively. Periodic boundary conditions were applied for the in-plane directions (*x*_1_ and *x*_2_); the top film surface is assumed to be stress-free, while the bottom surface of BFO is coherently strained by the substrate^63^. Electric biases were incrementally applied via the tip function described in the text.

## Additional information

**How to cite this article:** Li, Q. *et al.* Giant elastic tunability in strained BiFeO_3_ near an electrically induced phase transition. *Nat. Commun.* 6:8985 doi: 10.1038/ncomms9985 (2015).

## Supplementary Material

Supplementary Information Supplementary Figures 1-4, Supplementary Tables 1-2, Supplementary Notes 1-4 and Supplementary References.
