# Supplementary material for: Giant elastic tunability in strained BiFeO3 near an electrically induced phase transition
Source: Nat Commun. 2015 Nov 24;6:8985. doi: 10.1038/ncomms9985 (PMC4673877; doi:10.1038/ncomms9985)
Supplement: Supplementary Information — Supplementary Figures 1-4, Supplementary Tables 1-2, Supplementary Notes 1-4 and Supplementary References. [file ncomms9985-s1.pdf]

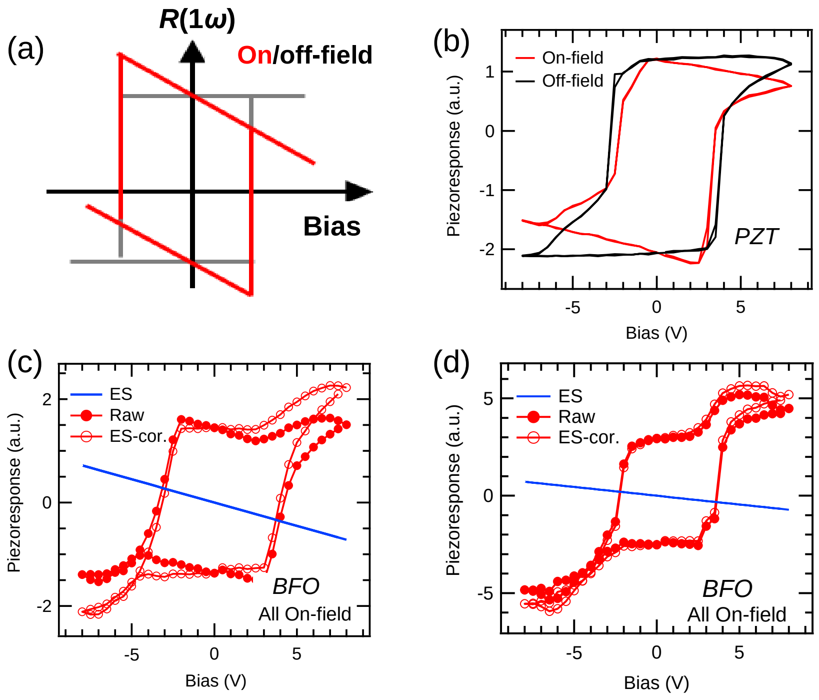

Supplementary Figure 1 | The electrostatic effect contribution. **a**, Schematic of the electrostatic contribution to measured bipolar BEPS (band-excitation piezoresponse spectroscopy) switching loops. **b**, An example of on/off-field switching loops measured on an epitaxial PZT thin film. **c**,**d**, BEPS on-field switching loops (Raw) of 50 nm $BiFeO_3/SrTiO_3$ thin film acquired when the tip was fresh (**c**) and relatively worn (**d**), along with the extracted electrostatic response (ES), and ES-corrected (ES-cor.) loops.

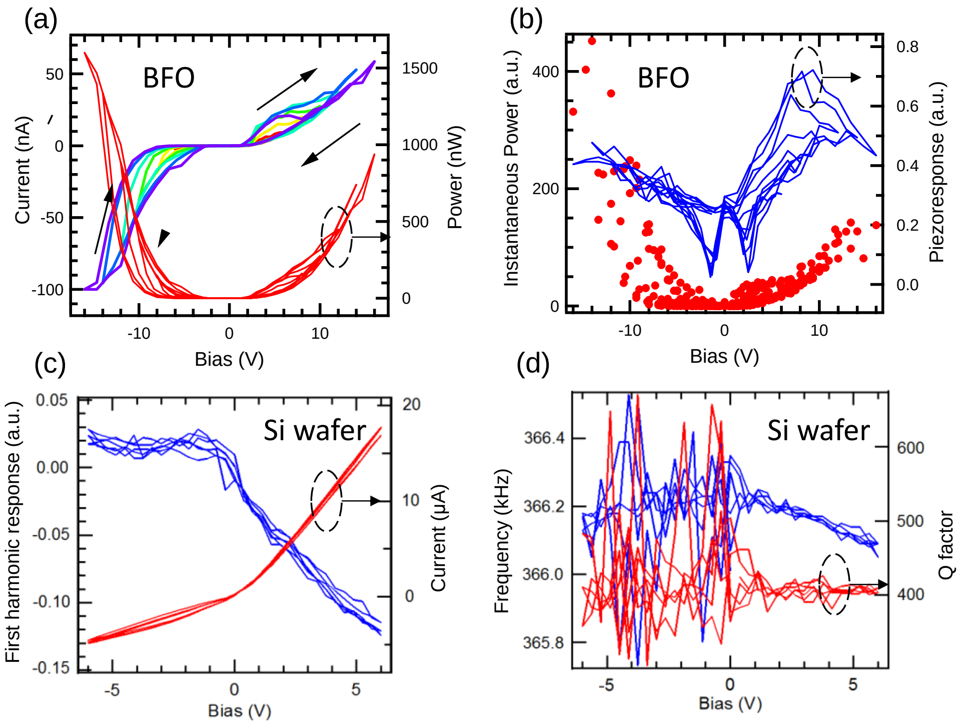

Supplementary Figure 2 | The Joule heating and thermal expansion effects. **a**,**b**, Current–voltage (*I*–*V*) curves and power (*VI*) loops (**a**) and instantaneous power and piezoresponse amplitude loops (**b**), all simultaneously measured with BEPS on 50 nm $BiFeO_3/SrTiO_3$ thin film. Arrows in **a** show the direction of *I*–*V* curves in correlation with Fig. 1e–g Main Text. **c**,**d**, *I*–*V* curves and first harmonic response loops (**c**) and resonance frequency and *Q* factor loops (**d**), measured on a *p*-type B-doped Si wafer. The negative half cycles in (**d**) are noisy due to the weak response spectra used for the simple harmonic oscillator (SHO) fitting.

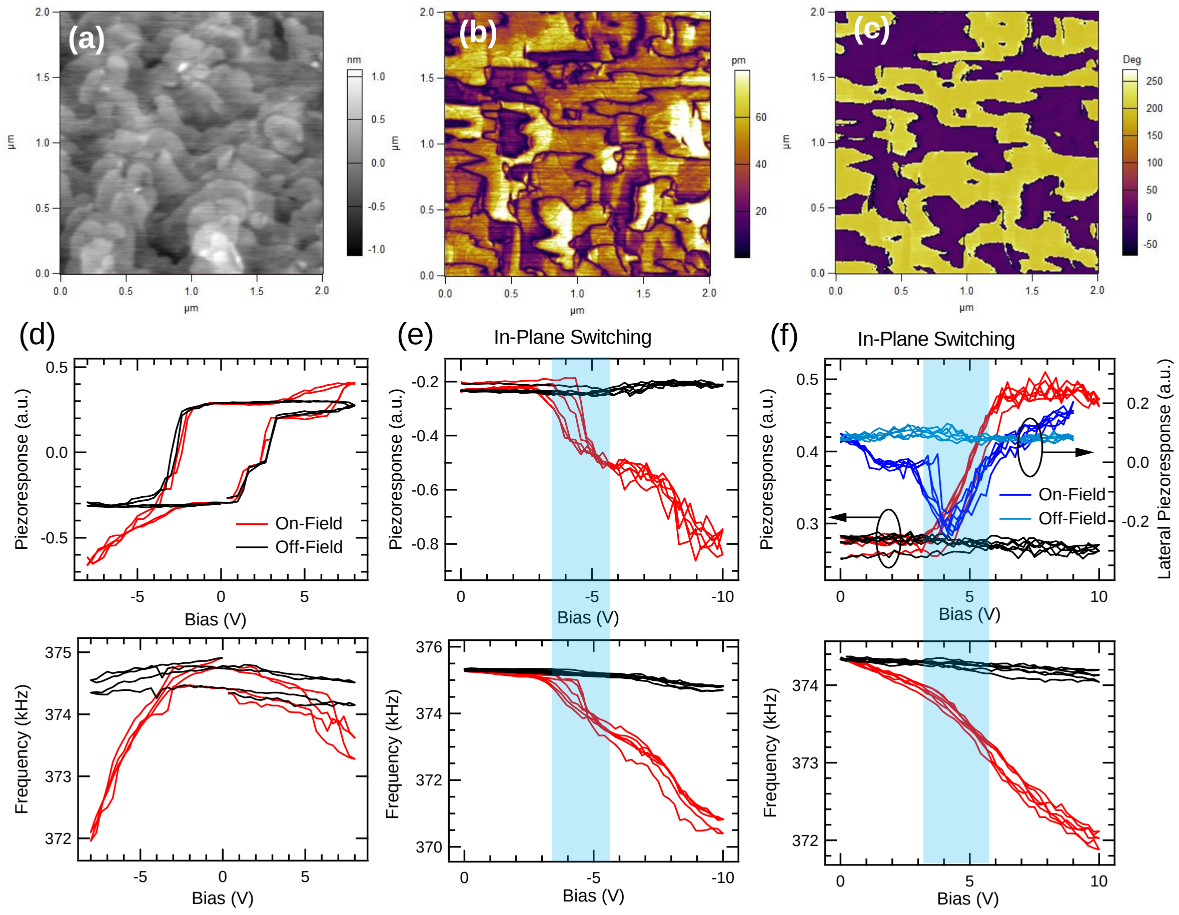

Supplementary Figure 3 | BEPS of 30 nm (001)-$BiFeO_3/DyScO_3$ epitaxial thin films. a–**c**, topography (**a**) and corresponding in-plane PFM domain images of the amplitude (**b**) and phase (**c**). **d**–**f**, Piezoresponse loops (*upper row*) and associated resonance frequency loops (*lower row*) measured in bipolar (**d**) and unipolar negative/positive (**e**/**f**) waveforms. All of them are vertical loops expect those explicitly annotated in **f**. The loops (including the in-plane switching loops) in **e**,**f** were measured at the same location with one or two pre-poling cycles in between them.

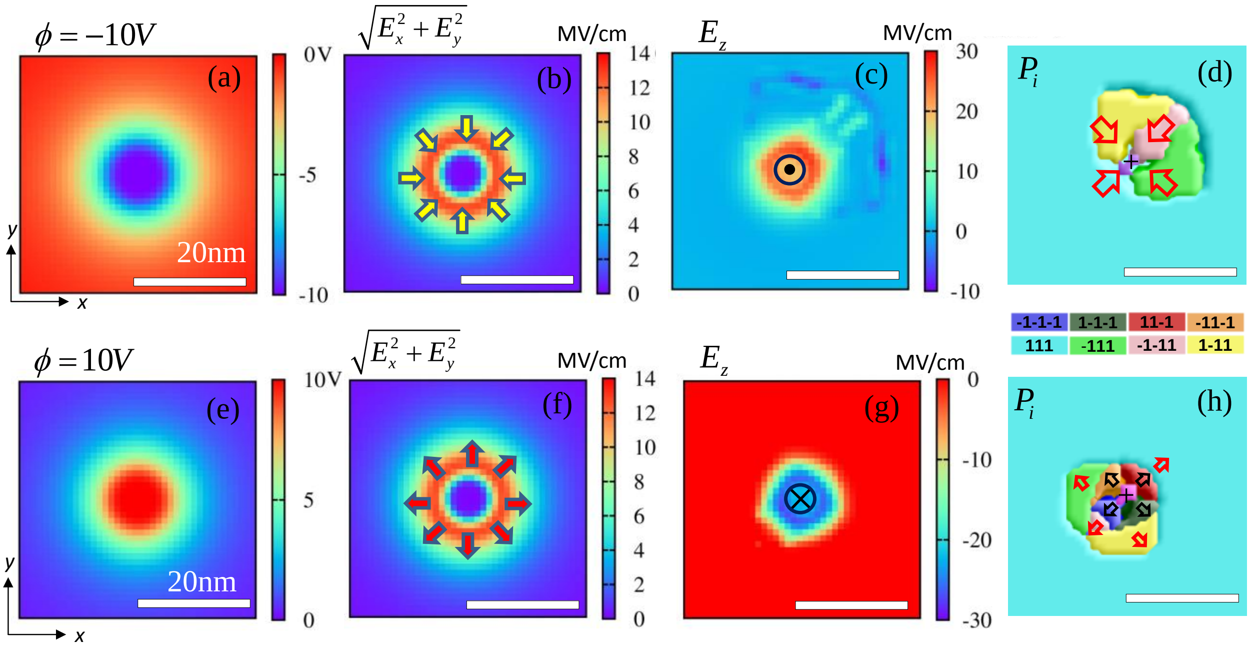

Supplementary Figure 4 | Anisotropic Domain switching of 50 nm (001)-BFO/STO thin films. 2D (*x*-*y*) profiles of tip potential (**a**,**e**), in-plane electric field magnitude (**b**,**f**), out-of-plane electric field component (**c**,**g**) and resultant domain configurations (**d**,**h**). **a**–**d** correspond to the parallel electric field case (–10V) and **e**–**h** the anti-parallel electric field case (+10 V). The arrows in **b**,**f** and the marks in **c**,**g** indicate the in-plane and out-of-plane electric field directions, respectively. The red arrows in **d**,**h** show the in-plane polarization directions of the R-phase domains with an upward P_Z component, and the black arrows in **h** show the in-plane polarization directions of the **R**-phase domains with a downward P_Z component. The “+” denotes the tip location in **d**,**h**.

Supplementary Table 1 | Calibration of the measured elastic properties. The free resonance frequency of the cantilever f_0 = 77.346 kHz and its stiffness k = 4.1 N/m.

**Materials**	**Pt** (average)	**Si** [001]	**$LiNbO_3$** (z-cut)	**$SrTiO_3$** [001]	**$BiFeO_3$** [001], 0 V
$\boldsymbol{Y}$ (GPa)	168	130	170[1]	270[2]	192[3]
$\boldsymbol{\nu}$	0.38	0.28	0.31	0.24	0.35
$\boldsymbol{f_C}$ (kHz)	—	367.1	372.5	377.7	375.2
$\boldsymbol{k^*}$ (N/m)	—	370	425	490	455
$\boldsymbol{Y^*}$ (GPa)	—	82.1	96.1	116.5	103
$\boldsymbol{Y^*_{Ref.}}$ (GPa)	—	—	97.5	116.6	106.3
$\boldsymbol{Y_{Calc.}}$ (GPa)	—	—	175	271	204

$\boldsymbol{Y}$: Young's modulus and $\boldsymbol{\nu}$: Poisson's ratio (both cited and/or recalculated from the literature values); $\boldsymbol{f_C}$: Measured contact resonance frequency; $\boldsymbol{k^*}$: Contact stiffness; $\boldsymbol{Y^*}$: Reduced Young's modulus; $\boldsymbol{Y^*_{Ref.}}$: Reduced Young's modulus with reference to Si by Supplementary Equation (3); $\boldsymbol{Y_{Calc.}}$ Calculated Young's modulus.

Supplementary Table 2 | Phase-field modeling parameters and energy coefficients.

**Landau coefficients**		**Elastic coefficients**		**Parameters in simulation**	
$\alpha_1 (10^8 C^{-2} m^2 N)$	-3.445	c_{11} (GPa)	302	T (°C)	25
$\alpha_{11} (10^9 C^{-4} m^6 N)$	2.604	c_{12} (GPa)	162	System size	128×128×64
$\alpha_{12} (10^9 C^{-4} m^6 N)$	-2.526	c_{44} (GPa)	69	Film (nm)	50
$\alpha_{111} (10^9 C^{-6} m^{10} N)$	1.92	$s_{11} (10^{-12} m^2 N^{-1})$	5.29	Substrate (nm)	10
$\alpha_{112} (10^8 C^{-6} m^{10} N)$	9.906	$s_{12} (10^{-12} m^2 N^{-1})$	1.85	a (nm)	3.0
$\alpha_{123} (10^9 C^{-6} m^{10} N)$	1.195	$s_{44} (10^{-12} m^2 N^{-1})$	14.7	γ (nm)	5.0
$\alpha_{1111} (10^8 C^{-8} m^{12} N)$	3.92	**Electrostrictive coefficients**		ε_s	0.5–2.5%
$\alpha_{1112} (10^7 C^{-8} m^{12} N)$	4.4	$Q_{11} (10^{-2} C^{-2} m^4)$	3.2	G_{ii}	0.3
$\alpha_{1122} (10^8 C^{-8} m^{12} N)$	-3.8	$Q_{12} (10^{-2} C^{-2} m^4)$	-1.6	κ_{ii}	50
$\alpha_{1123} (10^8 C^{-8} m^{12} N)$	8.0	$Q_{44} (10^{-2} C^{-2} m^4)$	2.015		

Supplementary Note 1. The extrinsic contribution to the measured on-field loops

Generally, the electrostatic effect contributes to measured first harmonic response, $R(1\omega)$, in PFM in proportion to the potential difference between the tip and sample surface via capacitive forces, according to the equation:

$$R(1\omega)_{ES} = -\alpha k_C^{-1} C_z' V_{ac}(V_{dc} - V_{sp}) \qquad (1)$$

where α is the sensitivity factor of the detection system, k_C contact stiffness, C_z' capacitance gradient of the tip–sample system, V_{sp} surface potential of the sample, and V_{ac}/V_{dc} applied a.c./d.c. voltages on the tip. For typical ferroelectric samples, the electrostatic interaction in the tip–sample junction is conservative; *i.e.*, the sample surface potential does not change significantly during BEPS measurements. Therefore, the electrostatic contribution to the on-field $R(1\omega)$ loop is a straight line with little hysteresis, as schematically shown in Supplementary Figure 1a. This is the reason why BEPS is predominantly performed in an off-field mode or only off-field loop data is paid attention to so as to minimize the electrostatic contribution. Supplementary Fig. 1b shows an example of bipolar switching loops measured on an epitaxial tetragonal-phase PZT thin film, which are in good agreement with the schematic. For our BFO thin film, the electromechanical response is found to be dominating compared to the electrostatic response even for on-field loops under our experiment conditions. Supplementary Fig. 1c,d shows two examples of on-field loops measured with the same parameters using a single tip, when it was relatively fresh (d) and significantly worn after long time usage (c). Note that electromechanical response in PFM is sensitive to the very tip apex; deterioration of the coatings therein reduces the effective local electrical fields and thus the measured response decreases (meanwhile the coercive biases increase and the loops appear to become broader). By contrast, the electrostatic forces mainly originate from the tip cone and cantilever beam and thus is far less sensitive to the wearing of tip apex coatings. The electrostatic contribution can be subtracted from the measured on-field loops based on the slopes shown on them, as illustrated in Suppl. Fig. 1c. In the cases of Suppl. Fig. 1d and most of the data presented in the Main Text, nevertheless, this subtraction can be of little significance for our analysis especially regarding the phase transition regions where the piezoresponse is markedly enhanced.

In BEPS, conduction current flowing through the tip–sample junction can cause Joule heating

that induces thermal expansion of the junction thereby potentially contributing the measured signals.[4] The Joule heating induced strain is proportional to the power (P) consumed on the tip–sample junction and expansion coefficients (β) of the sample as well as the tip, $x = \beta P$. In a general sense, assuming an ohmic conduction behavior with constant resistance R, $P = R I^2$. For on-field BEPS measurements, conduction current $I = I_{dc} + I_{ac} \sin(\omega t)$ due to both d.c. and a.c. applied voltages. This leads to a first harmonic response as a function of V_{dc}:

$$R(1\omega)_{JH} \propto 2\beta R I_{dc} I_{ac} \quad (2)$$

Analysis of this effect can be made from two aspects. First, let us consider the V_{dc} dependence (*i.e.*, loop shape) of this response based on the *I-V* curves simultaneously measured in BEPS. Since the conduction behavior here is no longer ohmic, we may consider the instantaneous power, *i.e.*, d*P*/d*V*, which should be approximately correlated with thermal strain response at a.c. modulation frequencies. Supplementary Figure 2a,b shows the *I–V* curves and BEPS amplitude loops from the same data set of Figure 1d–g in the Main Text. Obviously, no correlation exists between the measured conduction current and piezoresponse. Second, we measured a *p*-type Boron-doped Si wafer with a conductivity of 5–10 S m^{-1} under similar conditions. The conduction current in this case is two orders of magnitude higher than the BFO case, and measurable first harmonic response does exist and is in strong correlation with the *I–V* curves. This response, however, is much lower than the true piezoresponse of our BFO sample (*c.f.* Suppl. Fig. 2b,c). The contact resonance frequency of Si shows minute (~100 Hz) softening at high (positive) biases, presumably as a result of Joule heating taking place at the tip–sample junction. Note that although BFO has slightly lower heat capacity and higher thermal expansion coefficients than Si amounting to somewhat stronger Joule heating expansion effect in principle,[5] our comparative results still provide good indication of the contribution from this effect to the measured signals in BEPS. In addition, we also carried out finite element modeling of the Pt–BFO junction using Comsol Multiphysics 4.4 software based on the experimental parameters and measured conduction current. The temperature rises at the junction were found to be less than a few Kelvin which can be neglected for the main issues addressed in this work.

Supplementary Note 2. Quantitative analysis of the local elasticity of $BiFeO_3$

We chose three high quality commercially available single crystals of Si(001) wafer, PPLN (periodically-poled $LiNbO_3$, in *z*-cut) and $SrTiO_3$(001) as reference samples. The elastic moduli of these materials are expected to bracket those of $BiFeO_3$. We performed contact resonance atomic force microscopy (CR-AFM) measurements of these samples, using the photothermal excitation method as we recently reported.[6] An indentation force of 300 nN was set based on cantilever stiffness values calibrated with the standard thermal noise method, and was kept the same for all samples. To minimize uncertainties in contact radius arising from tip wearing during contact mode scanning, we acquired single point contact resonance spectra at over five random locations over each of the samples instead of acquiring CR-AFM images. For 50 nm $BiFeO_3$ (BFO) thin film, those locations were chosen away from surface corrugation regions (based on tapping mode images) that very likely correspond to domain walls. The resonance frequencies, f_C, measured on the reference samples showed variation less than ~500 Hz pertaining to their structural homogeneity as well as extremely smooth polished surfaces. For the BFO film, the f_C showed up to ~1 kHz maximum variation presumably largely due to different local curvatures in the topography. For the following analysis, we used the average measured f_C values and neglected the measurement errors.

Then we derived contact stiffness, k^*, from the measured f_C and free cantilever resonance frequency f_0 using cantilever dynamics models that we solved numerically based on the mathematic formalism of Rabe[7] and typical geometry factors of PPP-EFM cantilevers (see our previous results in Ref. [6]). With the contact stiffness values quantified, we were able to calculate elastic moduli of the samples based on the Hertzian contact mechanics model. The Hertzian model relates k_C with contact radius a_C and reduced Young's modulus Y^* as: $k^* = 2a_C Y^*$. Among them, Y^* is defined as:

$$\frac{1}{Y^*} = \frac{1-\nu_S^2}{Y_S} + \frac{1-\nu_T^2}{Y_T} \tag{3}$$

where ν_S, ν_T and Y_S, Y_T are the Poisson rations and Young's moduli of the sample and AFM tip (here, the values of the 25 nm conductive coating material, Pt, were used), respectively. The contact radius is related to the tip shape and applied force. Direct quantification of it from the Hertzian model could bring about large errors. Therefore, we followed a commonly used calibration

approach, according to the relation:

$$Y^* = Y^*_{\mathrm{Ref}} \left(k^* / k^*_{\mathrm{Ref}} \right)^m \tag{4}$$

where Y^*_{Ref} and k^*_{Ref} are the reduced Young's modulus and measured contact stiffness of the reference sample, respectively; *m* is tip geometry factor ranging from 1.5 for the case of hemispheric tip shape to 1 for a flat punch shape. The real tip geometry in practice is usually intermediate between these two ideal cases. We used *m* = 1.25 throughout our analysis as this value was found to yield the most self-consistent results for the three reference samples.

The elastic modulus of 50 nm BFO thin film in the pristine state (*i.e.*, zero applied biases) was calculated using the above method, resulting in Y_{11} = 204 GPa in good agreement with the literature (those values were derived from first principle calculations; note that our study is also one of not-many quantitative elastic measurements of $BiFeO_3$ at ultrasonic frequencies thus far). This value was then used as self-calibration for calculating the modulus change under applied tip biases based on the resonance frequency shifts observed at the same locations. The Poisson's ratio of $BiFeO_3$ was assumed unchanged during the bias-induced *R*–*T* phase transitions ($\nu = C_{12}/(C_{11}+C_{12})$, softening of the $C_{11}–C_{12}$ mode tends to increase the ν towards 0.5 thus meaning that the actual Y^{E} is smaller than our calculated results according to Supplementary Eq. 3; that is, our quantification of the softening effect is somewhat conservative). For single-point BEPS measurements, the contact radius does not change once the tip gets into stable contact with the samples. Otherwise it would be readily identified from the measured data; *e.g.*, discontinuous, irreversible frequency (upwards) jumps are usually observed when bias-induced topographic changes occur. The effects of d.c. bias-induced piezoelectric strains on the contact radius were also ruled out by comparison with other ferroelectric samples with similar levels of strain response, *e.g.*, $Pb(Zr,Ti)O_3$ (PZT) thin films.

Supplementary Note 3. Phase transition and softening behavior of (001)-$BiFeO_3$/$DyScO_3$

30 nm $BiFeO_3$ thin films with $SrRuO_3$ bottom electrodes were grown on (110)-cut $DyScO_3$ substrates using pulsed laser deposition under similar conditions as the 50 nm $BiFeO_3$/$SrTiO_3$ thin film primarily studied in this work. This BFO film has an extremely smooth surface with growth steps clearly discernible from the topography image; it also shows a homogeneous out-of-plane polarization component with downwards orientation (not shown) and well-defined in-plane domain

structures (Supplementary Fig. 3a−c). Suppl. Fig. 3d–f presents typical measured bipolar/unipolar BEPS loops. Similar bias-induced elastic softening and piezoresponse enhancement are observed on this sample, and the in-plane switching activities can be clearly identified from the (out-of-plane) piezoresponse loops. However, in comparison with the BFO/STO thin film, no obvious saturation/recovery behavior of the contact resonance frequency could be observed for this BFO film within up to ±10V bias windows in the experiments involving multiple fresh tips. Higher applied biases were found to significantly increase the propensity of irreversible changes in the topography and thus were not attempted further.

Supplementary Note 4. Anisotropic domain switching of R-phase BFO

The anisotropic growth of the switched domains is caused by the distribution of local electric fields. Under a negative tip bias of –10 V, the (inwards) in-plane electric field component switches the pristine $[111]_{\mathbf{R}}$ domain into three additional energetically favorable **R**-phase domains, $[1\text{-}11]_{\mathbf{R}}$, $[\text{-}1\text{-}11]_{\mathbf{R}}$ and $[\text{-}111]_{\mathbf{R}}$ (Supplementary Fig. 4a−d). Since the 71° switching is more favorable than the 109° switching, the $[1\text{-}11]_{\mathbf{R}}$ and $[\text{-}111]_{\mathbf{R}}$ appear earlier at smaller tip voltages and grow larger than the $[\text{-}1\text{-}11]_{\mathbf{R}}$ domain at –10 V. Note that the (upwards) out-of-plane electric field component, E_Z, is at its maximum beneath the tip apex where the in-plane field component is almost zero (*cf.* Suppl. Fig. 4b and c). In the case of positive tip bias +10 V, the E_Z is not strong enough in the region far away from the tip apex (outer-ring region) where, however, the (outward) in-plane electric field is strong enough to switch the pristine $[111]_{\mathbf{R}}$ domain into three additional upwards polarized R-phase domains ($[1\text{-}11]_{\mathbf{R}}$, $[\text{-}1\text{-}11]_{\mathbf{R}}$ and $[\text{-}111]_{\mathbf{R}}$) with the former two larger than the latter, similar to the parallel electric field situation. In the inner-ring region, the E_Z becomes large enough to switch the domain into four additional downwards polarized **R**-phase domains ($[\text{-}1\text{-}1\text{-}1]_{\mathbf{R}}$, $[1\text{-}1\text{-}1]_{\mathbf{R}}$, $[11\text{-}1]_{\mathbf{R}}$ and $[\text{-}11\text{-}1]_{\mathbf{R}}$). The presence of more domain variants in the positive bias case incurs more domain wall energies, which largely accounts for the smaller domain clusters formed than the negative bias case.

Supplementary References

1. Rivera, A., Garcia, G., Olivares, J., Crespillo, M. L. & Agulló-López, F. Elastic (stress–strain) halo associated with ion-induced nano-tracks in lithium niobate: role of crystal anisotropy. *J. Phys. D: Appl. Phys.* **44**, 475301 (2011).

2. Piskunov, S., Heifets, E., Eglitis, R. I. & Borstel, G. Bulk properties and electronic structure of $SrTiO_3$, $BaTiO_3$, $PbTiO_3$ perovskites: an ab initio HF/DFT study. *Computational Materials Science* **29**, 165−178 (2004).

3. Zhang, J. X. *et al.* Effect of substrate-induced strains on the spontaneous polarization of epitaxial $BiFeO_3$ thin films. *J. Appl. Phys.* **101**, 114105 (2007).

4. Kim, Y. *et al.* Nonlinear phenomena in multiferroic nanocapacitors: Joule heating and electromechanical Effects. *ACS Nano* **5**, 9104−9112 (2011).

5. Kallaev, S. N., Bakmaev, A. G. & Reznichenko, L. A. Thermal diffusion and heat conductivity of $BiFeO_3$ and $Bi_{0.95}La_{0.05}FeO_3$ multiferroics at high temperatures. *JETP Letters* **97**, 470−472 (2013).

6. Li, Q. *et al.* Probing local bias-induced transitions using photothermal excitation contact resonance atomic force microscopy and voltage spectroscopy. *ACS Nano* **9**, 1848−1857 (2015).

7. Rabe, U. in *Applied Scanning Probe Methods II* (eds Bharat Bhushan & Harald Fuchs) Ch. 2 (Springer, 2006).
